# Supplementary material for: Investigating the influence of physiologically relevant hydrostatic pressure on CHO cell batch culture
Source: Sci Rep. 2021 Jan 8;11:162. doi: 10.1038/s41598-020-80576-8 (PMC7794228; doi:10.1038/s41598-020-80576-8)
Supplement: Supplementary file 1 — Supplementary Information 1. [file 41598_2020_80576_MOESM1_ESM.zip › S1/IgG/Feb17 60 90 standard/standard/100ugml.pdf]

Data File C:\Users\P...\ChemStation\1\Data\TK_CHO_IgG 2019-02-17 17-28-56\005-6-100ug_ml.D
Sample Name: 100ug/ml

===

Acq. Operator : SYSTEM Seq. Line : 5
Sample Operator : SYSTEM
Acq. Instrument : HPLC-0039 Location : 6
Injection Date : 2/17/2019 5:47:39 PM Inj : 1
Inj Volume : 100.000 µl
Method : C:\Users\Public\Documents\ChemStation\1\Data\TK_CHO_IgG 2019-02-17 17-28-56
\CHO_IgG_LC.M (Sequence Method)
Last changed : 2/8/2019 3:17:23 PM by SYSTEM

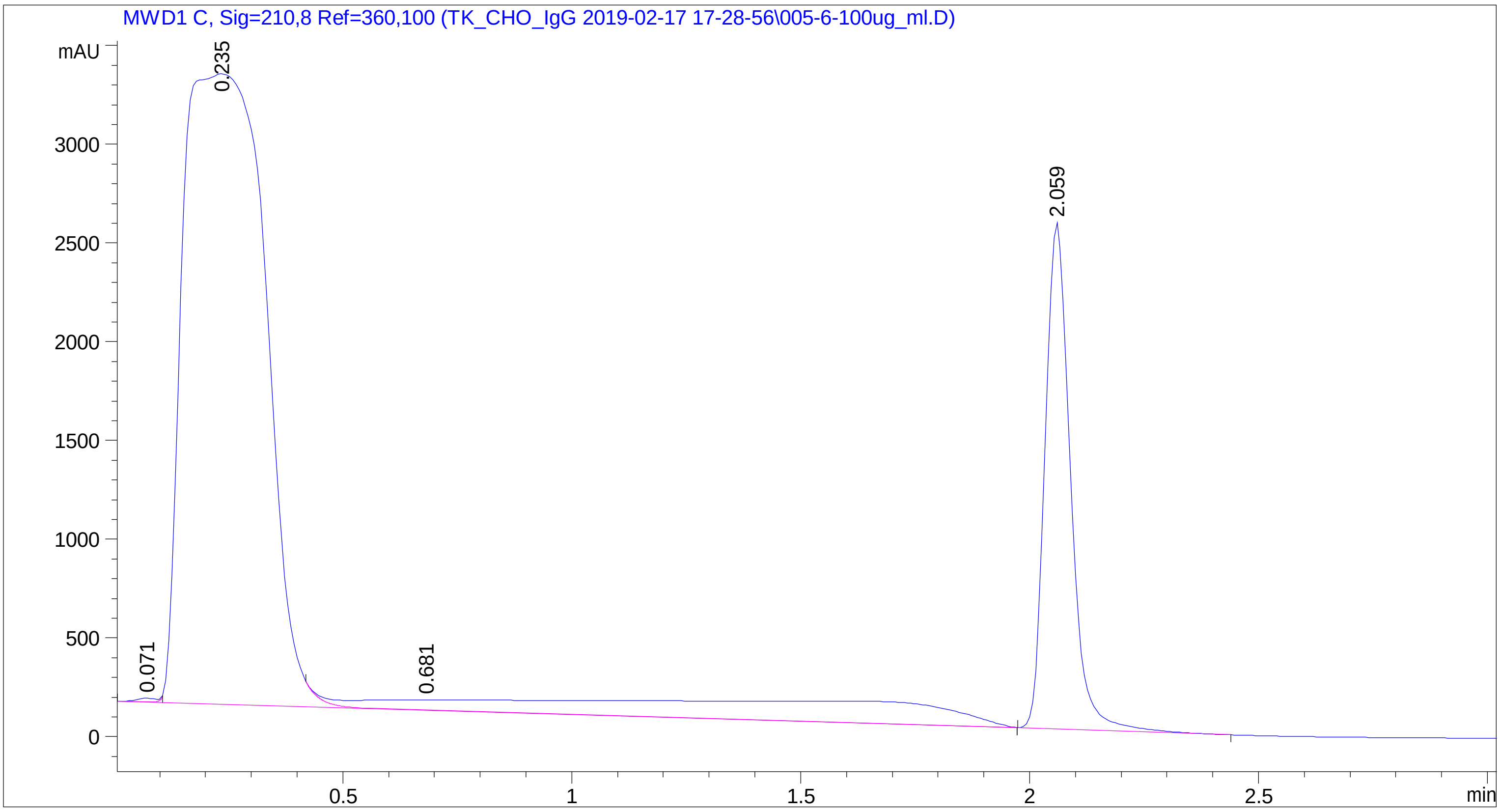

===

Area Percent Report

===

Sorted By : Signal
Multiplier : 1.0000
Dilution : 1.0000
Use Multiplier & Dilution Factor with ISTDs

Signal 1: MWD1 C, Sig=210,8 Ref=360,100

Peak #	RetTime [min]	Type	Width [min]	Area [mAU*s]	Height [mAU]	Area %
1	0.071	BV E	0.0463	54.99454	19.19748	0.0976
2	0.235	VV R	0.1830	3.96872e4	3192.17261	70.4598
3	0.681	VB E	1.5260	6678.75879	52.08541	11.8573
4	2.059	BBA	0.0591	9905.06445	2564.06055	17.5852

Totals : 5.63260e4 5827.51605

===

*** End of Report ***
